# Obesity and menopausal status impact the features and molecular phenotype of invasive lobular breast cancer

**DOI:** 10.1007/s10549-021-06453-8

**Published:** 2021-11-24

**Authors:** Harriet T. Rothschild, Mary Kathryn Abel, Anne Patterson, Kent Goodman, Amy Shui, Karen van Baelen, Christine Desmedt, Christopher Benz, Rita A. Mukhtar

**Affiliations:** 1 School of Medicine, University of California, San Francisco, CA 94143 USA; 2 Division of Surgical Oncology, Department of Surgery, University of California, San Francisco, CA 94143 USA; 3 Department of Surgery Biostatistics Core, University of California, San Francisco, CA 94143 USA; 4 Department of Oncology, Laboratory for Translational Breast Cancer Research, KU Leuven, B-3000 Leuven, Belgium; 5 Buck Institute for Research On Aging, Novato, CA 94945 USA

**Keywords:** Invasive lobular carcinoma, Metabolic syndrome, Oncotype RS, Menopausal status, BMI

## Abstract

**Purpose:**

We investigated the relationship between obesity, menopausal status, and invasive lobular carcinoma (ILC), the second most common histological subtype of breast cancer. Specifically, we evaluated the association between body mass index (BMI), metabolic syndrome, the 21-gene Oncotype Recurrence Score (Oncotype RS), and pathological features in patients with hormone receptor (HR)-positive, human epidermal growth factor receptor-2-negative ILC.

**Methods:**

The study cohort included 491 patients from a prospectively maintained institutional database consisting of patients with stage I-III, HR-positive ILC who underwent surgical treatment between 1996 and 2019.

**Results:**

Contrary to our expectations, we found that lower BMI was significantly associated with having higher Oncotype RS (18.9% versus 4.8%, *p* = 0.028) in post-menopausal patients, but was not related to tumor characteristics in pre-menopausal patients. Multivariate network analyses suggested a strong relationship between post-menopausal status itself and tumor characteristics, with lesser influence of BMI.

**Conclusion:**

These findings provide further insight into the recently appreciated heterogeneity within ILC and support the need for further investigation into the drivers of this disease and tailored treatment strategies.

## Introduction

Invasive lobular carcinoma (ILC) is the second most common histological subtype of breast cancer, accounting for 10%–15% of all invasive breast tumors [1]. ILC, of which the majority are estrogen receptor (ER) positive, appears to be particularly hormonally driven, as it is more strongly associated with early menarche, late menopause, and hormone replacement therapy use compared to invasive ductal carcinoma (IDC) [2–5]. Interestingly, as the rate of obesity has increased, the incidence of ILC among post-menopausal women has also increased, while that of invasive ductal carcinoma has remained stable [6].

While the complex relationship between obesity and breast cancer has been well studied, it remains incompletely understood. Obesity at breast cancer diagnosis has been shown to confer worse disease-free survival and overall survival in all breast cancer subtypes [7]. More specifically, increased adiposity has been implicated in breast cancer development in post-menopausal women and is associated with increased recurrence risk and mortality among patients with ER positive breast cancer [8–12]. These effects may be related to the production of estrogen and other mitogens by adipocytes, along with inflammation, vascularity, and fibrosis that stimulate breast tumor growth [13, 14]. Additionally, insulin resistance and systemic inflammation associated with obesity are thought to create a pro-tumoral environment, and indeed those with metabolic dysregulation described as the metabolic syndrome have been shown to have higher risk of breast cancer development [15]. Conversely, some studies find a protective effect of obesity on the risk of breast cancer development in pre-menopausal women, suggesting that the relationship between obesity and breast cancer differs by menopausal status [16].

In addition to impacting the risk of breast cancer development or recurrence, one study showed that obesity may be associated with different biological characteristics of ILC. Robinson et al. found that among 76 women with ILC, those with metabolic syndrome were significantly more likely to have high risk tumors as determined by the 70-gene signature MammaPrint score compared to those without metabolic syndrome [17]. Given that the development of ILC may be particularly tied to hormonal exposure, we aimed to evaluate associations between body mass index (BMI), metabolic syndrome, and ILC histopathological features in pre- versus post-menopausal women with early stage ILC. Additionally, we explored whether BMI and metabolic syndrome were associated with the 21-gene recurrence score (Oncotype RS) in a subset of patients with available scores.

## Methods

Following Institutional Review Board approval (17-23655, January 16, 2020), we retrospectively evaluated a prospectively maintained ILC database containing treatment and outcomes data for patients undergoing surgery at the University of California, San Francisco between January 1996 and September 2019. We included patients with stage I-III disease, and hormone receptor (HR) positive tumors. HR positivity was defined as having ≥ 1% either estrogen receptor (ER) or progesterone receptor (PR) staining on immunohistochemistry. ER and PR gene expression levels were also analyzed as continuous variables when data were available. We excluded cases with mixed ILC/IDC histology, human epidermal growth factor receptor-2 (HER2) overexpressing disease, and cases with missing BMI or menopausal status at the time of diagnosis. Histologic subtype was determined from review of surgical pathology reports, with ductal versus lobular histology determined with standard hematoxylin–eosin staining and selective use of E-cadherin staining. BMI was calculated as (weight kg)/((height m)^2) and categorized according to the World Health Organization classification (normal: < 25 kg/m^2^; overweight: 25–30 kg/m^2^; obese: ≥ 30 kg/m^2^). Metabolic syndrome was defined as having any 3 of the following 5 factors: obesity, hypertension, hypercholesterolemia, hypertriglyceridemia, and/or diabetes mellitus, as determined by recorded diagnosis or abnormal lab values in the electronic medical record [17]. Menopausal status at the time of breast cancer diagnosis was ascertained from oncology notes and was considered missing when not explicitly stated. Oncotype Recurrence Scores (RS) were recorded for the subset of patients for whom scores were obtained clinically. We analyzed RS both continuously and categorically, using the following risk categories (low: < 11; intermediate: 11–25; high: > 25).

Data were analyzed using the chi-square or Fisher’s exact tests, as appropriate, for categorical variables, and t-tests for continuous variables in Stata 14.2 (StataCorp LLC, College Station, TX, USA). Exploratory subgroup analyses were performed with subgroups selected based on scientific judgment. Hypothesis tests were two-sided, and the significant threshold was set to 0.05. In order to simultaneously examine the relationships between several co-occurring characteristics in our study sample, a Least Absolute Shrinkage and Selection Operator (LASSO) regularized partial correlation network analysis was performed with the polychoric correlation matrix method on the following variables: ER score, PR score, Oncotype RS (high versus intermediate/low), menopausal status (post-menopausal versus pre-menopausal), and weight status (BMI ≤ 25 versus BMI > 25). LASSO regularization increases the parsimony of the final network model, eliminating spurious connections. Network analysis was performed using the “qgraph” and “IsingFit” packages and “glasso” algorithm in R version 4.0.2. Data are presented in accordance with REporting recommendations for tumor MARKer prognostic studies (REMARK) recommendations [18].

## Results

### Overall cohort

**Fig. 1:**
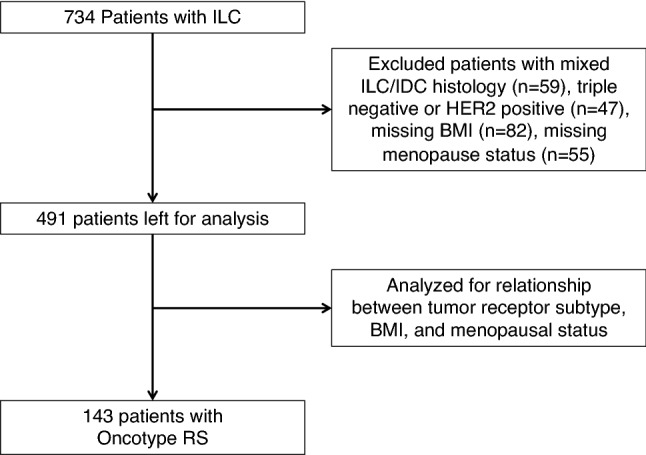
Flowchart depicting study design for analysis of ILC patients; *ILC* invasive lobular carcinoma, *IDC* invasive ductal carcinoma, and *RS* recurrence score

We identified 491 patients with HR-positive HER2-negative pure ILC, of whom 143 had available Oncotype RS, summarized in Fig. 1 and Table 1. The mean age was 59.8 years, ranging from 27 to 91, with 341 (69.5%) patients being post-menopausal and 150 (30.5%) being pre-menopausal. The mean BMI was 26.1 kg/m^2^ (standard deviation [SD] 5.8, range 14.8–61.4). Approximately half were of normal weight (*n* = 243, 50.6%), while 144 (29.9%) were overweight and 94 (19.5%) were obese. Metabolic syndrome was present in 84 patients (17.1%), with 105 (21.9%) having hypercholesterolemia, 56 (11.7%) having high triglycerides, 181 (37.1%) having hypertension and 46 (9.6%) having diabetes mellitus. As expected, post-menopausal patients were significantly older (mean age 64.9 years versus 48 years, *p* < 0.0001), were more likely to have a BMI above 25 (53.4% versus 40.0%, *p* = 0.017), and had higher rates of metabolic syndrome (21.7% versus 6.7%, *p* < 0.001) compared to pre-menopausal patients.

**Table 1:** Patient characteristics

Characteristics	Overall (*n* = 491)	Pre-menopausal (*n* = 150)	Post-menopausal (*n* = 341)	*P* Value
Age, mean (SD)	59.8 (11.6)	48 (5.5)	64.9 (9.7)	< 0.0001
Body mass index (BMI)				0.016
Normal weight	249 (50.7)	90 (60.0)	159 (46.6)	
Overweight	145 (29.5)	39 (26.0)	106 (31.1)	
Obese	97 (19.8)	21 (14.0)	76 (22.3)	
Metabolic syndrome present	84 (17.1)	10 (6.7)	74 (21.7)	< 0.001
ILC grade ^a^				0.315
1	145 (30.1)	51 (34.9)	94 (28.1)	
2	316 (65.7)	89 (61.0)	227 (67.8)	
3	20 (4.2)	6 (4.1)	14 (4.2)	
Hormone receptor subtype ^b^				< 0.001
ER + /PR +	373 (79.7)	131 (90.3)	242 (74.9)	
ER + /PR-	95 (20.3)	14 (9.7)	81 (25.1)	
ILC stage ^c^				0.059
I	315 (65.1)	87 (58.4)	228 (68.1)	
II	105 (21.7)	42 (28.2)	63 (18.8)	
III	64 (13.2)	20 (13.4)	43 (13.1)	

Data are expressed as n (%) unless otherwise specified. Total *n* = 491 unless otherwise specified. ILC, invasive lobular carcinoma; *ER*, estrogen receptor; *PR*, progesterone receptor

^a^Data available for 481

^b^Data available for 468

^c^Data available for 484

Most cases were stage I (*n* = 315, 65.1%), with 105 (21.7%) being stage II and 64 (13.2%) being stage III. Most tumors were grade 2 (*n* = 316, 65.7%), with 30.1% of patients having grade 1 and 4.2% having grade 3 disease. Tumor receptor subtype was ER positive/PR positive in 79.7%, with the remaining being ER positive/PR negative.

Of the 143 patients who had tumor profiling with Oncotype RS, 100 tumors (69.9%) were intermediate risk, 31 (21.7%) were low risk, and 12 (8.4%) were high risk (Table 2).

**Table 2:** Census of patients with Oncotype Recurrence Score (RS) by BMI and menopausal status

Categories	Overall	Pre-menopausal	Post-menopausal	*P *Value
Mean RS (SD) (*n* = 143)	15.4 (6.5)	13.8 (5.9)	16.7 (6.7)	0.006
Patients with BMI < 25 (*n* = 79)				0.028
Low risk RS	14 (17.7)	11 (26.2)	3 (8.1)	
Intermediate risk RS	56 (70.9)	29 (69.1)	27 (73.0)	
High risk RS	9 (11.4)	2 (4.7)	7 (18.9)	
Patients with BMI > 25 (*n* = 64)				0.714
Low risk RS	17 (26.6)	6 (27.3)	11 (26.2)	
Intermediate risk RS	44 (68.8)	16 (72.7)	28 (66.7)	
High risk RS	3 (4.6)	0 (0.0)	3 (7.1)	

Oncotype RS risk categories defined as: low < 11; intermediate 11–25; high > 25. Data are expressed as *n* (%) unless otherwise specified

### Tumor characteristics

There was no significant difference in tumor grade or stage by menopausal status; however, there was a significant difference in tumor receptor subtype. Post-menopausal women were significantly more likely to have ER positive/PR negative ILC versus ER positive/PR positive ILC compared to pre-menopausal women (25.1% versus 9.7%, *p* < 0.001).

Overall, we found no significant association between BMI and tumor receptor subtype. However, among the non-obese group (BMI < 25 kg/m^2^), post-menopausal patients were significantly more likely to have ER positive/PR negative tumors compared to pre-menopausal patients (27.6% versus 10.4%, *p* < 0.001). Among those with obesity, menopausal status was not associated with tumor receptor subtype.

There was no association between metabolic syndrome and tumor receptor subtype. However, those with metabolic syndrome were significantly more likely to have grade 3 tumors than those without metabolic syndrome, although grade 3 disease was uncommon overall (9.5% versus 3% grade 3, respectively, *p* = 0.005).

### Oncotype recurrence score

**Fig. 2:**
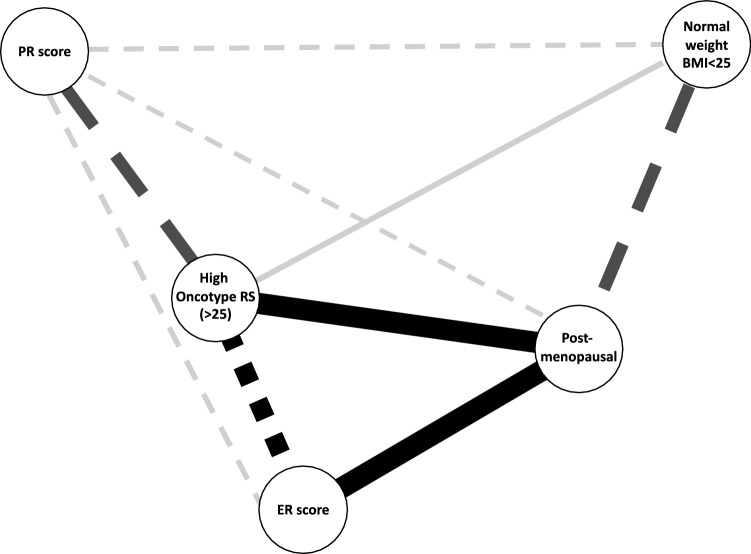
Network analysis with solid lines indicating a positive relationship and dashed lines indicating a negative relationship. Thicker lines and darker gradient designate stronger relationships. *ER* estrogen receptor, *PR* progesterone receptor, *RS* recurrence score

Of the 491 patients in the cohort, 143 (29%) had Oncotype RS available for analysis. Mean RS was significantly higher in post-menopausal women than pre-menopausal women (RS of 16.7 versus 13.8, *p* = 0.006). Overall, BMI was not associated with Oncotype RS category. However, the distribution of Oncotype RS varied significantly when both BMI and menopausal status were considered (Fig. 2). Among those with normal weight, post-menopausal patients were significantly more likely to have high RS tumors than pre-menopausal patients (18.9% versus 4.8%, *p* = 0.028). Among those with overweight/obesity, there was no significant association between menopausal status and RS category.

Similarly, the relationship between Oncotype RS and metabolic status was seen among post-menopausal patients only. In the post-menopausal cohort, mean RS was significantly lower in those with metabolic syndrome compared to those without metabolic syndrome (mean RS 13.6 versus 17.6, *p* = 0.0297). There was no significant association between metabolic syndrome and RS among pre-menopausal patients in this cohort.

### Multivariate network analysis

**Fig. 3:**
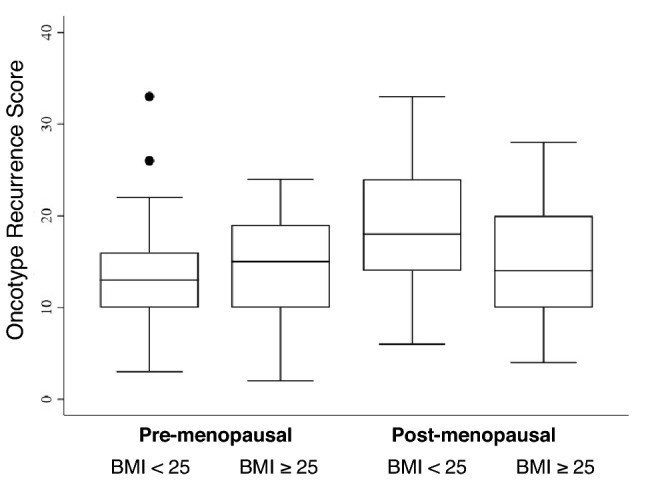
Box plot of Oncotype RS by menopausal status and BMI. In pre-menopausal women, mean RS did not differ significantly by BMI category (13.6 in normal weight versus 14.1 in overweight/obese, *p* = 0.76). However, in post-menopausal women, those with normal BMI had significantly higher RS than those with overweight/obesity (18.9 versus 14.8, *p* = 0.0072). Normal weight is defined as BMI < 25 kg/m^2^, while overweight/obese is BMI ≥ 25 kg/m^2^

To understand the relationships between these multiple co-occurring variables, we utilized multivariate network analysis including continuous ER score, continuous PR score, Oncotype RS (high versus intermediate/low), BMI (< 25 versus ≥ 25), and menopausal status. In this network (Fig. 3), the strongest relationships were noted between Oncotype RS and both ER score and PR score, and between ER score, Oncotype RS, and menopausal status. Higher ER score and higher PR score were strongly related to intermediate/low Oncotype RS. Being post-menopausal was related to having a high Oncotype RS. Additionally, being post-menopausal was strongly related to having BMI ≥ 25 and with having higher ER score, while it was weakly related to having lower PR score. Those with normal BMI were strongly correlated with having pre-menopausal status. Normal BMI was weakly related to both lower PR score and high Oncotype scores. Overall in this network, menopausal status and ER score had the greatest impact on relationships between variables, while BMI category and PR score had the least impact.

## Discussion

In this study of women with ER positive, HER2 negative, pure ILC, we found that BMI and metabolic syndrome impact ILC tumor biology as determined by histological grade, tumor receptor subtype, and Oncotype RS. These relationships, however, differ by menopausal status. Among non-obese patients, ER positive/PR negative tumors were significantly more common in post-menopausal versus pre-menopausal patients. However, among those with obesity, tumor receptor subtypes were similar by menopausal status. Interestingly, we found that post-menopausal patients had a significantly higher proportion of ER positive/PR negative tumors than pre-menopausal women, particularly among non-obese patients. Similarly, those with metabolic syndrome had a higher proportion of higher-grade tumors in post-menopausal women.

Our findings highlight the complex relationships between obesity/metabolic syndrome and breast cancer tumor biology by menopausal status in ILC, a particularly hormonally driven tumor type. The multivariate network analysis we performed illustrates the complex interplay that these variables have on ILC tumor biology.

Prior studies have found that high BMI is associated with less aggressive tumor types in the post-menopausal setting, and more aggressive tumor types in the pre-menopausal setting [19, 20]. Consistent with this, we found that overweight/obese post-menopausal patients in our study had lower RS compared to post-menopausal patients with normal BMI. Others have suggested that post-menopausal women with breast cancer are more likely to develop PR negative tumors and that circulating levels of estrogen are protective against breast cancer development in obese post-menopausal patients [21, 22]. Our findings raise the possibility that the hormonal pathogenesis and estrogenic drive behind ILC differs by menopausal status, possibly due to more local production of estrogen from higher breast adiposity in post-menopausal women relative to the greater systemic ovarian production of estrogen in pre-menopausal women.

Others have shown that obesity and metabolic syndrome result in worse outcomes, potentially suggesting that these are associated with more aggressive tumor types. Additionally, metabolic syndrome has been shown to be associated with more aggressive ILC as determined by MammaPrint scores. While we did not find a significant association between metabolic syndrome and tumor type overall, surprisingly we found a significant association between metabolic syndrome and lower RS in the post-menopausal group. This finding is contradictory to prior work and could be related to differences in Oncotype versus MammaPrint, with PR expression being more highly weighted in the Oncotype RS. We hypothesize that the higher rates of PR expression in overweight/obese post-menopausal women resulted in lower RS, and that the strong association between overweight/obesity and metabolic syndrome led to this finding. Additionally, the rate of metabolic syndrome in our overall population was low, and may have prevented us from finding an association between metabolic syndrome and tumor subtype in the pre-menopausal population. Ascertaining metabolic syndrome from review of medical records and diagnostic codes is a less sensitive method than others, for example, measuring waist to hip ratio as an indicator of visceral adiposity. Less precision in our designation of metabolic syndrome in combination with low rates of obesity and metabolic syndrome overall in this cohort could account for differences in our findings. A similar analysis of Oncotype RS in a cohort of 269 patients with breast cancer also found that post-menopausal patients had higher RS and lower PR expression than pre-menopausal patients, which is consistent with the higher rates of PR negativity seen in our post-menopausal cohort [23]. However, they found that higher BMI was associated with higher RS, whereas we found the opposite. Since histologic subtype was not reported, this raises the possibility that the relationship between higher BMI and lower RS in post-menopausal patients is unique to ILC, but further study is needed.

It is also important to comment on the utility of Oncotype and MammaPrint in the setting of ILC. In a recently published large prospective trial of 353 patients with ILC and 2232 patients with non-lobular breast cancer, there was a threefold lower prevalence of high RS results for patients with ILC, but the 5-year disease-free survival between ILC and non-lobular breast cancer was similar [24]. These findings raise the possibility of differential prognostic significance of molecular assays in ILC versus non-lobular tumors, at least perhaps in the first five years.

Recent data implicate lipid metabolism in ILC, with ILC expressing more fatty acid binding protein than invasive ductal carcinoma, and factors related to lipid metabolism being involved in endocrine resistance in ILC [25–27]. While ILC was previously thought to be a homogenous tumor type, newer data identify subtypes within ILC with molecular assays showing distinct gene expression subtypes among ILC tumors. In addition, it is known that E-cadherin (*CDH-1*) loss is a diagnostic feature of ILC with more increased methylation associated with greater cell invasiveness and metastatic potential [28, 29]. Leptin, a cytokine produced by adipocytes, was found to increase E-cadherin expression in an in vivo mouse model [30]. That study’s demonstration of the influence of obesity on tumor epigenetics potentially supports the finding of a relationship between BMI and RS in ILC. The differential impact of BMI metabolic factors to ILC development needs further investigation and could result in new approaches to prevention and treatment.

Strengths of our study include access to a unique, well annotated, and continually updated institutional ILC database that contains characteristics of over 700 patients with ILC including 143 with corresponding Oncotype RS. However, the retrospective nature of this study has inherent weaknesses, including lack of Oncotype RS testing on every tumor. Patient or provider bias could have influenced whether Oncotype RS testing was performed, and this is unaccounted for in our analysis. Also, we extrapolated obesity from BMI, which does not accurately reflect visceral adiposity. Additionally, factors such as age at menarche, parity, and use of hormonal replacement therapy were not available in this dataset.

## Conclusions

Overall, our findings suggest that BMI and menopausal status impact tumor characteristics in ILC, raising the possibility that the pathogenesis of ILC may differ by menopausal status. These findings support the recently appreciated heterogeneity within ILC and suggest that further investigation into the drivers of this disease and more tailored prevention and treatment strategies are needed.

## Data Availability

The data supporting all tables in this published article are not publicly available to protect patient privacy, but can be accessed from the corresponding author on request. Data will be made available to authorized researchers who have obtained Institutional Review Board (IRB) approval from their own institution and from the University of California, San Francisco IRB.

## Abbreviations

BMI: Body Mass Index
ER: Estrogen Receptor
HER2: Human Epidermal Growth Factor Receptor-2
HR: Hormone Receptor
IDC: Invasive Ductal Carcinoma
ILC: Invasive Lobular Carcinoma
LASSO: Least Absolute Shrinkage and Selection Operator
PR: Progesterone Receptor
RS: Recurrence Score
